# A Hybrid Model for Forecasting Sunspots Time Series Based on Variational Mode Decomposition and Backpropagation Neural Network Improved by Firefly Algorithm

**DOI:** 10.1155/2018/3713410

**Published:** 2018-10-14

**Authors:** Guohui Li, Xiao Ma, Hong Yang

**Affiliations:** School of Electronic Engineering, Xi'an University of Posts and Telecommunications, Xi'an, Shaanxi 710121, China

## Abstract

The change of the number of sunspots has a great impact on the Earth's climate, agriculture, communications, natural disasters, and other aspects, so it is very important to predict the number of sunspots. Aiming at the chaotic characteristics of monthly mean of sunspots, a novel hybrid model for forecasting sunspots time-series based on variational mode decomposition (VMD) and backpropagation (BP) neural network improved by firefly algorithm (FA) is proposed. Firstly, a set of intrinsic mode functions (IMFs) are obtained by VMD decomposition of the monthly mean time series of the sunspots. Secondly, the firefly algorithm is introduced to initialize the weights and thresholds of the BP neural network, and a prediction model is established for each IMF. Finally, the predicted values of these components are calculated to obtain the final predict results. Comparing BP model, FA-BP model, EMD-BP model, and VMD-BP model, the simulation results show that the proposed algorithm has higher prediction accuracy and can be used to forecast the time series of sunspots.

## 1. Introduction

Sunspot is the most basic and obvious activity in the solar activity, and the sunspot numbers are an indicator of the total solar activity level. Time-series data of sunspot are considered to be nonlinear, nonstationary, and chaotic, which are widely used to evaluate the effectiveness of nonlinear time-series models [1]. Sunspot has a very significant impact on the Earth's magnetic field, climate, geology, animals, and plants and also endangers the living environment of human beings. Therefore, the observation and prediction of the sunspot numbers are of great significance. At present, with a large amount of long-term historical data, researchers have proposed a variety of prediction methods to forecast years, monthly mean, and peak of sunspots, such as chaos theory [2, 3], various neural networks, and their optimization algorithms. Among them, Ding et al. [4] used BP neural network to predict monthly mean of smoothed sunspot area. Zhao et al. [5] used RBF neural network to predict the smoothed monthly mean sunspot numbers, but the prediction error was gradually enlarged with the prolongation of forecasting time. Artificial neural networks inherently slowed down the learning speed and have poor fault tolerance and easily fell into local minima. These defects have not been well improved, thus the prediction accuracy cannot be further improved. The rise of intelligent optimization algorithms has greatly improved these shortcomings of artificial neural networks. Guan et al. [6] used quantum-behaved particle swarm optimization to optimize the weights and thresholds of BP neural networks to predict sunspot numbers. Li et al. [7, 8] used genetic algorithms to optimize BP neural networks to predict short-term traffic flow chaotic time series. The experimental results show that the optimized models are better.

Empirical mode decomposition (EMD), wavelet decomposition, and other methods provide an idea for processing nonlinear signals. In references [9, 10], EMD has been applied to the decomposition prediction of nonlinear time series, but the EMD has the effect of mode mixing and point effects [11]. The introduction of ensemble empirical mode decomposition (EEMD) [12] and variational mode decomposition (VMD) [13] has improved the mode mixing to some extent. Especially VMD transforms signal decomposition into nonrecursive variational mode decomposition mode. Its number of components is also less than EMD and EEMD, and it shows better noise robustness.

In recent years, hybrid models of EMD, EEMD, and VMD combined with artificial neural networks have been widely used in the field of prediction [9, 10, 14, 15]. The hybrid forecasting models formed by combining VMD with other algorithms have been successfully applied in many fields, such as financial time series [16], stock price evaluation [17], wind speed [18], and so on. Lahmiri [16] developed a new model of VMD and general regression neural network (GRNN) to analyze and predict economic and financial time series. In reference [17], the VMD and BP neural network were combined to predict the stock price well. In reference [18], the VMD was used to predict the wind speed sequence and then greatly reduced the data complexity and improved the prediction accuracy. Therefore, this paper proposes a novel hybrid forecasting model based on VMD and firefly algorithm (FA) to optimize BP neural network (FA-BP) and applies the model to the forecast of sunspot numbers.

## 2. Basic Theory

### 2.1. Variational Mode Decomposition

VMD is an effective signal decomposition method. Its overall framework is a variational problem [13, 19], which is different from the EMD of circular filtering. Its each mode is assumed to be a finite bandwidth with a different center frequency and the goal is to minimize the sum of the estimated bandwidths for each mode. The algorithm can be divided into the structure and solution of variational problem. The detailed description is as follows.

#### 2.1.1. The Structure of Variational Problem

Assume the original signal *f* is decomposed into *K* modal functions *u*_*k*_(*t*)(*k*=1,2,…, *K*), such that the variational problem can be described as a constrained variational problem. The problem is targeted at minimizing the sum of the estimated bandwidth for each mode, and the constraint is that the sum of modes is equal to the input signal *f*, as follows:

(1) $$\begin{array}{c} \left\{\begin{array}{c} \underset{\left\{u_{k}\right\},\left\{\omega_{k}\right\}}{\min}\left\{\overset{K}{\underset{k=1}{\sum }}{\left\|\partial_{t}\left[\left(\delta \left(t\right)+\frac{j}{\pi t}\right)u_{k}\left(t\right)\right]e^{-j\omega_{k}t}\right\|}_{2}^{2}\right\},\\ \text{s}.\text{t}.\overset{K}{\underset{k=1}{\sum }}u_{k}=f. \end{array}\right. \end{array}$$

#### 2.1.2. The Solution of Variational Problem

- The above constrained variational problem can be changed into a nonbinding variational problem by introducing a quadratic penalty factor *C* and Lagrange multipliers *θ*(*t*). Where *C* guarantees the reconstruction accuracy of the signal and *θ*(*t*) maintains the rigor of the constraint. The augmented Lagrange is denoted as

(2) $$\begin{array}{c} L\left(\left\{u_{k}\right\},\left\{\omega_{k}\right\},\theta \right)=C\overset{K}{\underset{k=1}{\sum }}{\left\|\partial_{t}\left[\left(\delta \left(t\right)+\frac{j}{\pi t}\right)u_{k}\left(t\right)\right]e^{-j\omega_{k}t}\right\|}_{2}^{2}+\;{\left\|f\left(t\right)-\overset{K}{\underset{k=1}{\sum }}u_{k}\left(t\right)\right\|}_{2}^{2}+\langle \theta \left(t\right),f\left(t\right)-\overset{K}{\underset{k=1}{\sum }}u_{k}\left(t\right)\rangle . \end{array}$$

(b) The alternate direction multiplier method (ADMM) is used to solve the saddle points of the above variational problem, and then the *u*_*k*_^*n*+1^, *ω*_*k*_^*n*+1^, and *θ*^*n*+1^ are updated alternately (*n* represents the number of iterations), which is given by

(3) $$\begin{array}{c} {\hat{u}}_{k}^{n+1}\left(\omega \right)=\frac{\hat{f}\left(\omega \right)-\sum_{k=1}^{K}{\hat{u}}_{k}\left(\omega \right)+\left(\hat{\theta }\left(\omega \right)/2\right)}{1+2C{\left(\omega -\omega_{k}\right)}^{2}}, \end{array}$$

(4) $$\begin{array}{c} \omega_{k}^{n+1}=\frac{\int_{0}^{\infty }\omega {\left|{\hat{u}}_{k}\left(\omega \right)\right|}^{2}d\omega }{\int_{0}^{\infty }{\left|{\hat{u}}_{k}\left(\omega \right)\right|}^{2}d\omega }, \end{array}$$

(5) $$\begin{array}{c} {\hat{\theta }}^{n+1}\left(\omega \right)={\hat{\theta }}^{n+1}\left(\omega \right)+\tau \left[\hat{f}\left(\omega \right)-\overset{K}{\underset{k=1}{\sum }}{\hat{u}}_{k}^{n+1}\left(\omega \right)\right]. \end{array}$$

(c) Given a discriminant accuracy *e* > 0, the convergence condition of the stop iteration is as follows:

(6) $$\begin{array}{c} \overset{K}{\underset{k=1}{\sum }}\frac{{\left\|{\hat{u}}_{k}^{n+1}-{\hat{u}}_{k}^{n}\right\|}_{2}^{2}}{{\left\|{\hat{u}}_{k}^{n}\right\|}_{2}^{2}}<e. \end{array}$$

The specific process of the VMD algorithm is summarized as follows:

1. Initialize {*u*_*k*_^1^}, {*ω*_*k*_^1^}, ${\hat{\theta }}^{1}$ and *n*.
2. Update the value of $\left\{{\hat{u}}_{k}^{n+1}\right\}$, {*ω*_*k*_^*n*+1^} and ${\hat{\theta }}^{n+1}$ according to Equations (3)–(5).
3. Judge whether or not *u*_*k*_ meets the convergence condition (6), and repeat the above steps to update parameters until the convergence stop condition is satisfied.
4. The corresponding modal subsequences are obtained according to the given modal number.

### 2.2. FA-BP Model

#### 2.2.1. Backpropagation Neural Network

**Figure 1:**
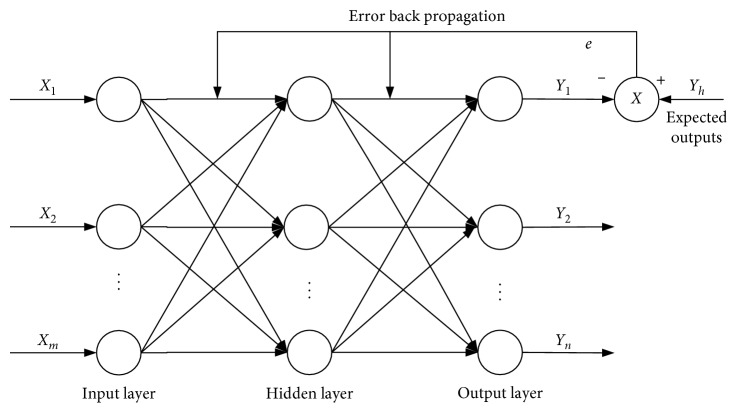
The structure of 3-layer BP neural network.

Backpropagation (BP) network is a multilayered feedforward neural network trained by error backpropagation. It has good self-organizing learning ability and can implement any nonlinear mapping from input to output [20, 21]. The network prediction model mainly realizes the training process through the forward propagation of the input signal and the backpropagation of the error signal. It can parallelize large-scale data and has a certain degree of robustness and fault tolerance. The typical structure of three-layer BP neural network is shown in Figure 1.

In Figure 1, *X*_1_, *X*_2_,…, *X*_*m*_ is input value of BP neural network, *Y*_1_, *Y*_2_,…, *Y*_*n*_ is output value of BP neural network, and *Y*_*k*_ is the expected output. The input vectors are propagated layer by layer from input layer, hidden layer, and output layer. The number of input layer nodes and output layer nodes are determined by the features and dimensions of training samples, respectively. The number of hidden layer nodes needs to be selected artificially according to the situation. Setting the transfer function between the layers as used sigmoid function, the output of the *j*th node of the hidden layer is

(7) $$\begin{array}{c} H_{j}=\frac{1}{1+\exp\left(-\sum_{i=1}^{n}\omega_{ij}x_{i}-a_{j}\right)},\;j=1,2,\ldots ,l, \end{array}$$

where *ω*_*ij*_ is the connection weight of the *i*th node of the input layer to the *j*th node of the hidden layer, *a*_*j*_ is the threshold of the *j*th node of the hidden layer, and *l* is the number of hidden layer nodes. The hidden layer output *H*_*j*_ is further passed back to get the *k*th node of the output layer as

(8) $$\begin{array}{c} Y_{k}=\frac{1}{1+\exp\left(-\sum_{j=1}^{l}\omega_{jk}H_{j}-b_{k}\right)},\;k=1,2,\ldots ,n, \end{array}$$

where *ω*_*jk*_ is the connection weight of the *j*th node of the hidden layer to the *k*th node of the output layer, *b*_*k*_ is the threshold of the *k*th node of the output layer, and *m* is the number of output layer nodes. According to the network prediction output *Y*_*k*_ and the expected output *Y*_*h*_, calculate the network prediction error *E*:

(9) $$\begin{array}{c} E=Y_{h}-Y_{k}. \end{array}$$

BP neural network corrects the weights and thresholds of all layers along the direction of error reduction and repeatedly modifies the weights and thresholds until the algorithm converges to obtain satisfactory error precision.

#### 2.2.2. Firefly Algorithm

Firefly algorithm (FA) was developed by Cambridge scholar Yang [22]. It is a swarm intelligence optimization algorithm based on the idealized behavior of the flashing characteristics of fireflies. Comparing with other optimization algorithms, the main advantages of firefly algorithm are simple structure, less adjustment parameters, and better spatial search capability [23]. Therefore, the application field of the algorithm is also quite extensive such as function optimization, path planning, resource management, and so on [24–27].

In this algorithm, the brightness of a firefly and mutual attractiveness are the key factors that determine the firefly's movement. The fitness value of the firefly's position determines its brightness. The brighter the brightness, the better its position. Attractiveness and brightness are related. The higher brightness of the fireflies has a stronger attraction, so other fireflies of lower brightness approach in this direction. Through the interaction of brightness and attractiveness, fireflies will eventually gather in the highest brightness position to achieve the optimization of objective function. In order to facilitate the establishment of the algorithm model, the following definition is given:

*Definition 1*. The relative brightness of a firefly:

(10) $$\begin{array}{c} I=I_{0}e^{-\gamma r_{ij}^{2}}, \end{array}$$

where *I*_0_ is the original light intensity which is related to the objective function value, *γ* is the light absorption coefficient, generally defined as a constant 1, and *r*_*ij*_ is the Cartesian distance between firefly *i* and firefly *j*. The expression is as follows:

(11) $$\begin{array}{c} r_{ij}=\left\|x_{i}-x_{j}\right\|=\sqrt{\overset{d}{\underset{k=1}{\sum }}{\left(x_{i,k}-x_{j,k}\right)}^{2}}. \end{array}$$

*Definition 2*. The attractiveness of a firefly is as follows:

(12) $$\begin{array}{c} \beta =\beta_{0}e^{-\gamma r_{ij}^{2}}, \end{array}$$

where *β*_0_ is the maximum attractiveness which represents the degree of attraction at the location of the maximum brightness. Equation (12) describes the property that the attractiveness of fluorescence emitted by fireflies to other individuals decreases with distance and the absorption coefficient of the propagation medium. *β*_0_ can be taken as 1 for most application problems. The value of *γ* has a great influence on the performance of the algorithm. Theoretically, we can take *γ* ∈ [0, *∞*), but in practice we take usually *γ* ∈ [0.1, 10].

*Definition 3*. Updated formula for the position of firefly *i* moved by firefly *j* is as follows:

(13) $$\begin{array}{c} x_{i}\left(t+1\right)=x_{i}\left(t\right)+\beta_{ij}\left(r_{ij}\right)\left[x_{j}\left(t\right)-x_{i}\left(t\right)\right]+\alpha \left(\mathrm{rand}-0.5\right), \end{array}$$

where *t* is the iteration number of the algorithm, *x*_*i*_, *x*_*j*_ are the spatial positions of the firefly *i* and *j*, *α* is the step factor, and taking the constant at [0,1], rand is the random factor that obeys the uniform distribution on [0,1].

The process of firefly optimization algorithm is as follows:

1. Initialize the basic parameters of FA and set the number of firefly population *m*, maximum attractiveness *β*_0_, light absorption coefficient *γ*, step size *α*, the maximum number of iterations max  *T*, and search accuracy *ε*.
2. Randomly initialize the location of fireflies and calculate the target value of fireflies which is used as their maximum brightness *I*_0_.
3. Calculate the relative brightness *I* and attractiveness *β* of the fireflies in the group by (10) and (12) and determine the direction of the firefly's movement according to the relative brightness.
4. According to Equation (13), to update the spatial location of fireflies, the firefly at the best position is randomly perturbed by *α*(rand − 0.5) to prevent premature convergence and fall into the local optimal solution.
5. According to the location of the updated firefly, recalculate the brightness of fireflies.
6. Turn on (7) when the search accuracy or the maximum number of searches is satisfied. Otherwise, the number of searches will be increased by 1 and turn (3) to the next search.
7. Output the global extreme point and the optimal individual value.

#### 2.2.3. FA-BP Prediction Model

In the BP neural network, the two kinds of training parameters of weight matrices *ω*_*ij*_, *ω*_*jk*_ and thresholds *a*_*j*_, *b*_*k*_ have significant influences on the prediction accuracy. The basic principle of optimizing FA-BP neural network is to use the better global search ability of FA to optimize the topology, connection weights, and thresholds of neural network. Considering the network parameters of BP neural network as firefly individuals, the fitness function of firefly individuals and the training error output of the corresponding network model form a linear relationship in the process of network model training:

(14) $$\begin{array}{c} f\left(x_{i}\right)=-E_{x_{i}}. \end{array}$$

According to the above equation, it shows that the smaller the training error, the greater the individual fitness value. In the firefly algorithm, the greater the fitness value, the greater the brightness, indicating that firefly individuals perform better on the optimization problem. By searching for the best individual, which is the optimal network parameter, the firefly position is constantly updated, and then the weights and thresholds are substituted into the BP neural network to predict the sample. Specific steps are as follows:

1. Determine the neural network structure according to the input sample characteristics and output requirements.
2. Initialize BP neural network, determine the number of neurons in each layer, and calculate the weight and the threshold number.
3. Consider weights and thresholds as individual fireflies and initialize firefly algorithm parameters.
4. Carry out the firefly algorithm iterative update process and search for the best individual fitness.
5. The optimal individuals are transmitted back to BP neural network for training and prediction with test data.

**Figure 2:**
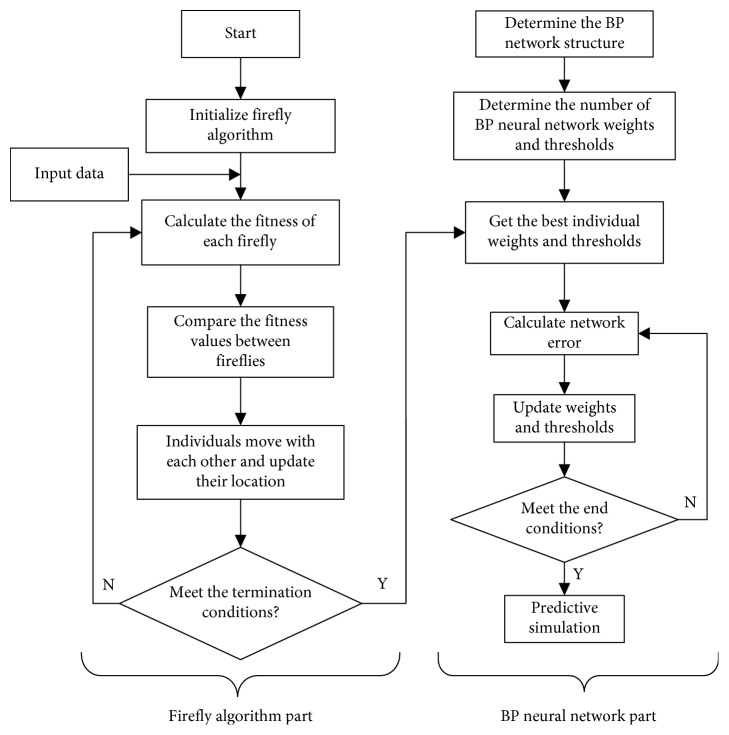
The flowchart of FA-BP prediction model.

The flowchart of FA-BP prediction model is shown in Figure 2.

### 2.3. Hybrid VMD-FA-BP Forecasting Model

In this section, the proposed VMD-FA-BP model is established for the monthly mean of sunspots forecasting. The basic structure of the hybrid forecasting method is as follows:

1. *First decomposition*. The time series of monthly mean of sunspots are decomposed by VMD into IMFs with different frequencies.
2. *Component prediction*. The data of each IMF component is divided into training samples and predicting samples and normalize input/output samples. Then, the FA-BP prediction model is established, respectively, to obtain the predicted value of each component.
3. *Reconstruct the predicted value*. The predicted values of each IMF component *P*_1_, *P*_2_,…, *P*_*n*_ are summed up to obtain the final prediction result.

**Figure 3:**
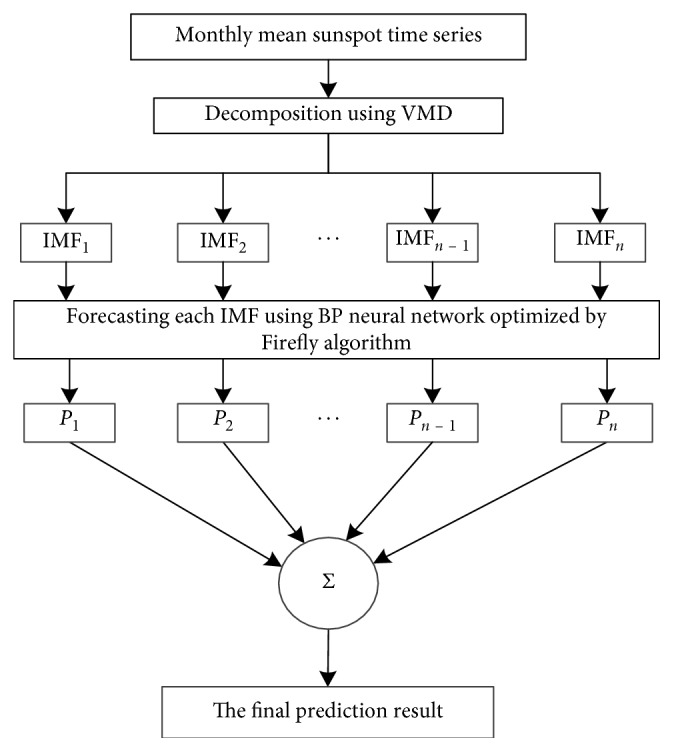
The flowchart of VMD-FA-BP model.

The flowchart of VMD-FA-BP model is shown in Figure 3.

## 3. Data Simulation and Analysis

### 3.1. Data Decomposition Preprocessing

**Figure 4:**
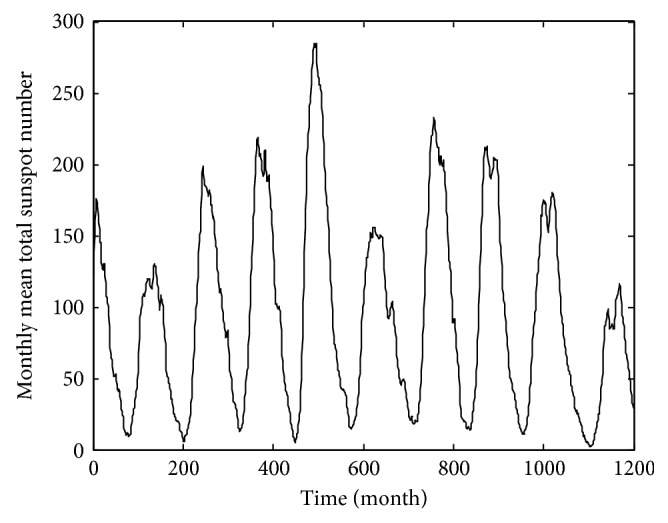
Monthly mean sunspot time series.

**Figure 5:**
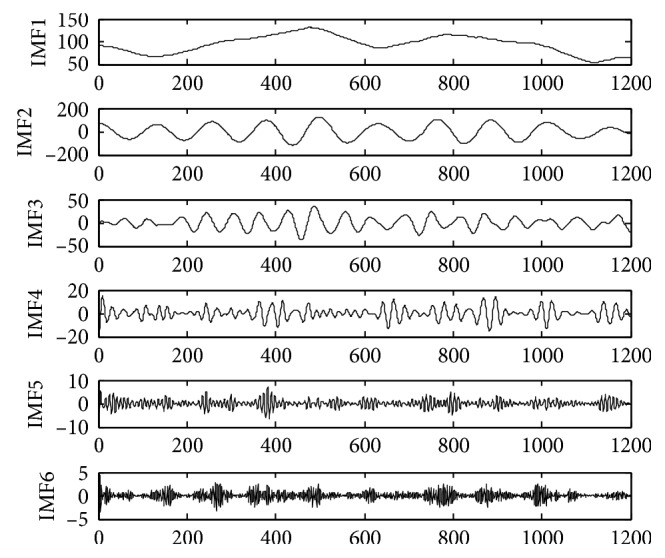
Decomposition results by VMD.

**Figure 6:**
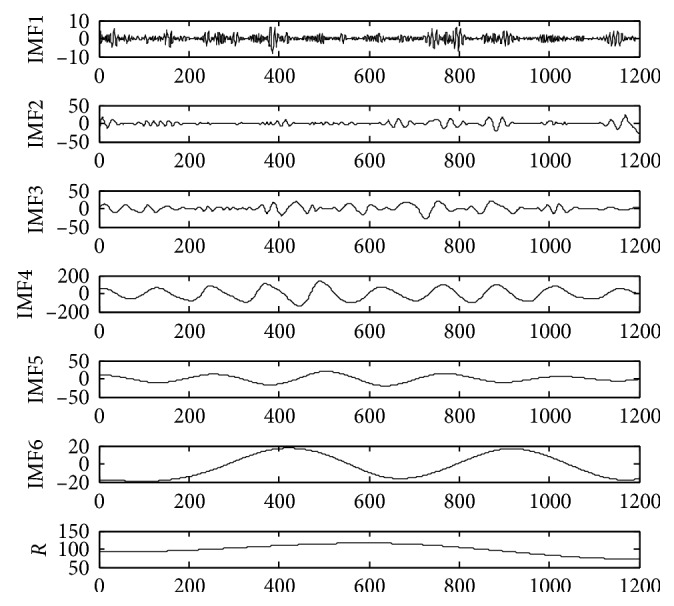
Decomposition results by EMD.

The time series of monthly mean sunspots data comes from the official website of the solar influence data analysis center (SIDC). The experiment selects the observation data from January 1917 to December 2016 as the experimental sample, a total of 1200 data. The time-series diagram is shown in Figure 4. Taking into the randomness of the monthly mean of sunspots, direct prediction there will be a greater error. In order to improve the accuracy of prediction, the data complexity needs to be reduced. The original sequence is decomposed to generate multiple subsequences by VMD. Set the number of subsequences *K* before proceeding with VMD decomposition. However, we can see from the previous test that for the average monthly sequence of sunspots, the following subsequences tend to be similar when *K* > 6, so we choose *K* = 6 in this paper. The original data VMD decomposition is shown in Figure 5. The same data sample is also decomposed by EMD as shown in Figure 6.

As shown in Figures 5 and 6, the time series of sunspots are decomposed into 6 IMFs by VMD, and 6 IMFs and 1 residual component are obtained by EMD decomposition. From the decomposition results, it can be seen that the IMF3 component decomposed by the VMD method is most similar to the original signal waveform. The waveform distortion is small, and the trend of the high-frequency component variation is relatively stable. So, it is good for prediction. Although the low-frequency component fluctuates greatly, the prediction error is limited. Because the final VMD forecast result is an accumulation of each IMF's forecast result, the characteristics of the VMD decomposition result help to improve the prediction accuracy. However, the IMFs discomposed by EMD have different characteristics. End-point effects occur during the decomposition process. Large fluctuations occur at both ends of the IMF component and affect the entire component sequence continuously. The error would be more serious and the decomposition results would be seriously distorted, especially for low-frequency IMF components. Moreover, the drastic fluctuations of the IMF will lead to large errors in the final forecast based on the EMD. Therefore, IMFs decomposed by VMD are more suitable for the establishment of hybrid forecasting model than IMFs decomposed by EMD [28–30].

### 3.2. Performance Standards of Prediction Accuracy

This study uses the following three error indicators to verify the validity and practicability of the proposed prediction model: mean absolute error (MAE), root mean squared error (RMSE), and mean absolute percentage error (MAPE). The error of prediction value is quantified by using the performance indexes of MAE, RMSE, and MAPE. The smaller the value, the better the prediction accuracy. These formulas are as follows:

(15) $$\begin{array}{c} \text{MAE}=\frac{1}{n}\overset{n}{\underset{i=1}{\sum }}\left|\hat{x}\left(t\right)-x\left(t\right)\right|,\\ \text{RMSE}=\sqrt{\frac{1}{n}\overset{n}{\underset{i=1}{\sum }}{\left[\hat{x}\left(t\right)-x\left(t\right)\right]}^{2}},\\ \text{MAPE}=\frac{1}{n}\overset{n}{\underset{i=1}{\sum }}\left|\frac{\hat{x}\left(t\right)-x\left(t\right)}{x\left(t\right)}\right|, \end{array}$$

where $\hat{x}\left(t\right)$ is forecast data and *x*(*t*) is original data. In order to avoid the errors caused by the difference and randomness of each prediction result, this paper averages the results of 10 predictions, that is, the error value of the final prediction.

### 3.3. Data Prediction Processing and Results Analysis

All the simulation models in this paper are completed in MATLAB 2014a. The first 900 of the 1200 samples in the experimental sample are taken as the training samples, and the last 300 are used as the prediction samples. The BP neural network prediction model adopts the network structure of 5-8-1 and adopts the same structure for the FA-BP network prediction model, that is, the number of nodes in the input layer is 5, the number of nodes in the input layer is 8, and the number of nodes in the input layer is 1. There are 5 × 8+8 × 1=48 weights, 8+1=9 thresholds. The individual code length of the firefly algorithm is 48+9=57. The parameters are set as follows: the number of fireflies *m*=30, the maximum attraction *β*_0_=1, the light absorption coefficient *γ*=1, the step factor *α*=0.2, and the maximum number of iterations max  *T*=100. The above parameter settings and sample data are applied to all model tests in this article to ensure fair and efficient comparisons between different forecast models.

**Figure 7:**
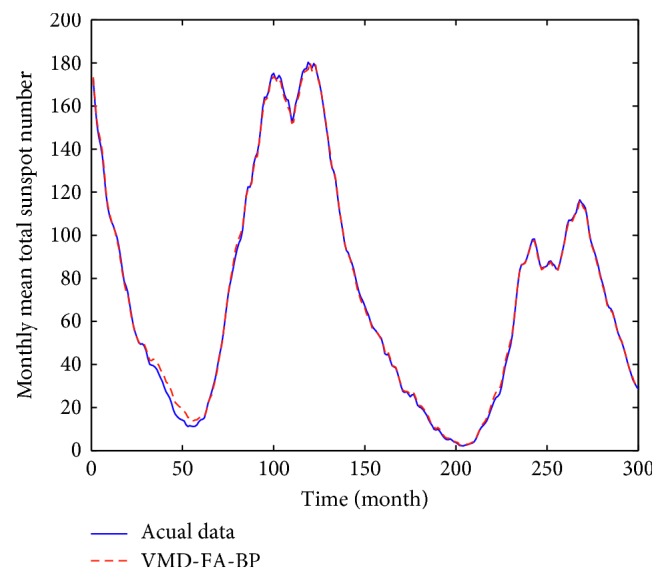
Prediction results of VMD-FA-BP model.

**Figure 8:**
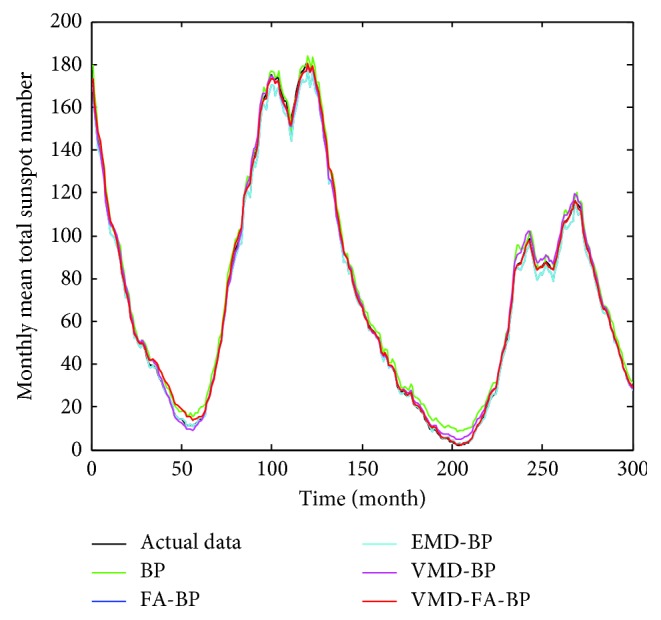
Predicted results of sunspot numbers for each model.

**Figure 9:**
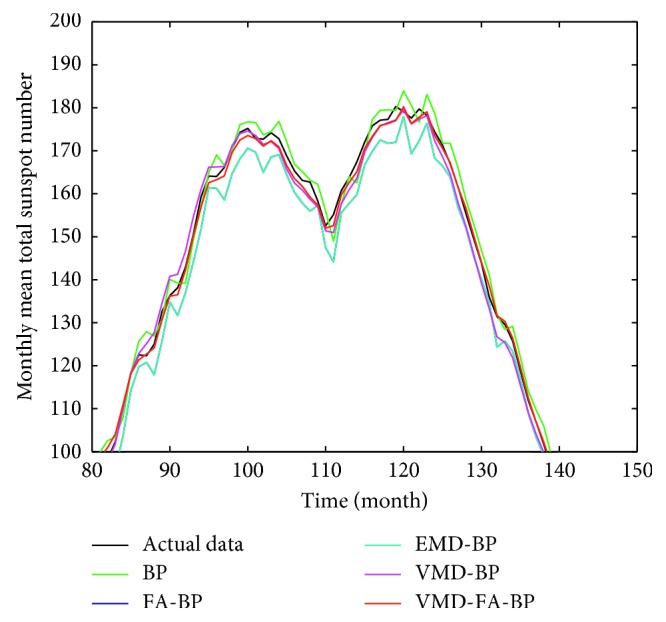
Local predicted result graphs of sunspot numbers for each model.

**Figure 10:**
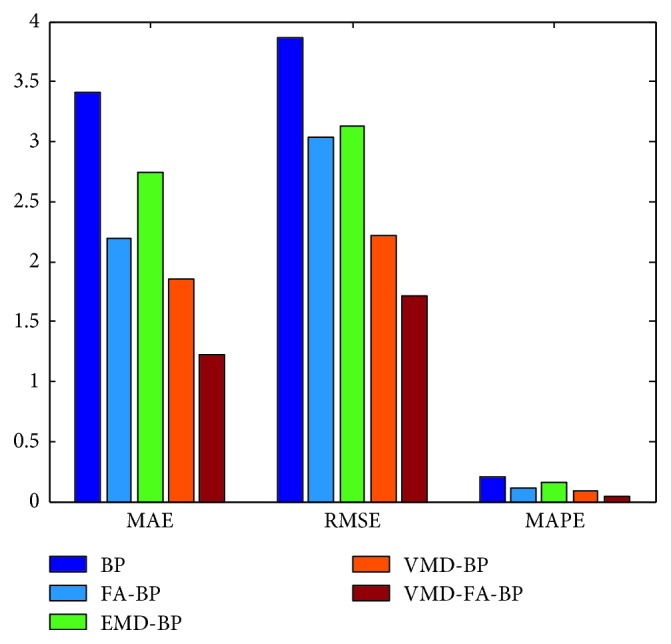
Error graphics of different models.

According to the above settings, each IMF after VMD decomposition is predicted and reconstructed to obtain the final prediction value. The prediction result is shown in Figure 7. It can be seen from the figure that the blue line represents the actual monthly mean of sunspots and the red line represents the predicted value of the hybrid forecasting model. It can be seen that the VMD-FA-BP model proposed in this paper is good for fitting the original data and can predict the monthly mean of sunspots very well. In order to facilitate comparison, BP neural network, FA-BP, EMD-BP, and VMD-BP prediction models are used to predict the same sunspot monthly mean time series, as shown in Figure 8. The partial enlargement is shown in Figure 9. While the forecasting performance evaluation results are displayed in Table 1 and Figure 10, respectively.

It can be seen from Table 1 and Figure 9 that the three error indicators of the VMD-FA-BP prediction model proposed in this paper are less than other prediction models, where MAE = 1.2208, RMSE = 1.7117, and MAPE = 0.0435, and its forecasting accuracy is improved by one order of magnitude compared with other models.

This obvious change is chiefly due to the following reasons. First, as a new adaptive multiresolution technique, the VMD is more robust to analyze noisy signals such as sunspot data than EMD and reduces the mode mixing existed in the EMD, making each IMF used for the following predictions better. Secondly, the processing time required by VMD, EMD, and BP neural network is significantly short. When processing monthly mean sunspot time series, the VMD and EMD computational time is 10s30 and 12s26, respectively. The BP neural network takes on average 0.95 s for learning and testing, compared with the traditional autoregressive moving average model in [14, 28], which takes more time for modeling and forecasting (more than 5 min) and saves a lot of time and improves the prediction efficiency and accuracy. Then, the firefly algorithm is a feasible and effective group intelligent optimization algorithm with high optimization precision and convergence speed. The optimal initial weights and thresholds of the BP neural network are obtained by using the firefly optimization algorithm, and the FA-BP model is applied to predict each IMF, and the obtained effect is better than a single BP model. Finally, the predicted values of each IMF are accumulated to obtain the final predicted values, thus the best prediction accuracy is shown in all the models of Table 1.

Hybrid model is more beneficial for modeling and improving prediction accuracy by using the advantages of each single model. It is proved that the variational mode decomposition combined with BP neural network prediction model using firefly algorithm in this paper can predict the variation trend of the monthly mean of sunspots time series well, which is a good prediction model.

## 4. Conclusions

In this paper, aiming at the problem of forecasting the monthly mean time series of sunspots, a hybrid forecasting model based on variational mode decomposition and firefly algorithm to optimize BP neural network is proposed. Firstly, a set of intrinsic mode functions (IMFs) are obtained by VMD decomposition of the monthly mean of the sunspots. Secondly, a prediction model is established for each IMF to improve the prediction accuracy. In order to overcome the defects of the slow learning speed and easy to fall into local minimum in BP neural network, firefly algorithm is used to optimize the BP neural network. The optimal weights and thresholds are obtained. The training samples of the network are trained, and then the test samples are predicted. Finally, the predicted values of these components are summed to obtain the final prediction result, and the predicted value is compared with the real value to calculate the error. The experimental results show that the combination method proposed in this paper can be used to predict the monthly mean of sunspots and improve the prediction accuracy and reduce the error compared with other models. Therefore, the model proposed in this paper has a good reference value for forecasting actual problems accurately such as short-time traffic flow and other time series.

## Figures and Tables

**Table 1:** Comparison of various methods for error analysis of forecasting samples.

Models	Error indicators		
	MAE	RMSE	MAPE
BP	3.4037	3.8647	0.2065
FA-BP	2.1931	3.0317	0.1072
EMD-BP	2.7458	3.1338	0.1596
VMD-BP	1.8497	2.2107	0.0867
VMD-FA-BP	1.2208	1.7117	0.0435

## Data Availability

The smoothed monthly mean sunspot number time series in this paper comes from the data analysis center of the Sun Observatory of Belgium (http://sidc.oma.be/silso/datafiles). It records the sunspot data from 1749 to the present. In particular, the data analysis center significantly revised the number of sunspots on July 1, 2015. The data in this paper were derived from the revised dataset.
